# Shotgun metagenomic analysis of microbial communities from the Loxahatchee nature preserve in the Florida Everglades

**DOI:** 10.1186/s40793-019-0352-4

**Published:** 2020-01-21

**Authors:** Briana S. Abraham, Deniz Caglayan, Natalie V. Carrillo, Matthew C. Chapman, Claire T. Hagan, Skye T. Hansen, Ralph O. Jeanty, Alexander A. Klimczak, Marcos J. Klingler, Thomas P. Kutcher, Sydney H. Levy, Angel A. Millard-Bruzos, Thomas B. Moore, David J. Prentice, Matthew E. Prescott, Richard Roehm, Jordan A. Rose, Mulan Yin, Ayumi Hyodo, Kathleen Lail, Christopher Daum, Alicia Clum, Alex Copeland, Rekha Seshadri, Tijana Glavina del Rio, Emiley A. Eloe-Fadrosh, Jonathan B. Benskin

**Affiliations:** 1 Boca Raton Community High School, Boca Raton, FL 33486 USA; 2 Department of Ecosystem Science and Management, Texas A&M University, College Station, TX 77843 USA; 3 Department of Energy, Joint Genome Institute, Berkeley, CA 94720 USA

**Keywords:** Shotgun metagenomics, Methane production, Nitrogen fixation, Everglades, Soil metagenome, Prokaryotes

## Abstract

**Background:**

Currently, much is unknown about the taxonomic diversity and the mechanisms of methane metabolism in the Florida Everglades ecosystem. The Loxahatchee National Wildlife Refuge is a section of the Florida Everglades that is almost entirely unstudied in regard to taxonomic profiling. This short report analyzes the metagenome of soil samples from this Refuge to investigate the predominant taxa, as well as the abundance of genes involved in environmentally significant metabolic pathways related to methane production (nitrogen fixation and dissimilatory sulfite reduction).

**Methods:**

Shotgun metagenomic sequencing using the Illumina platform was performed on 17 soil samples from four different sites within the Loxahatchee National Wildlife Refuge, and underwent quality control, assembly, and annotation. The soil from each sample was tested for water content and concentrations of organic carbon and nitrogen.

**Results:**

The three most common phyla of bacteria for every site were Actinobacteria, Acidobacteria, and Proteobacteria; however, there was variation in relative phylum composition. The most common phylum of Archaea was Euryarchaeota for all sites. Alpha and beta diversity analyses indicated significant congruity in taxonomic diversity in most samples from Sites 1, 3, and 4 and negligible congruity between Site 2 and the other sites. Shotgun metagenomic sequencing revealed the presence of biogeochemical biomarkers of particular interest (e.g., *mrcA, nifH, and dsrB)* within the samples. The normalized abundances of *mcrA*, *nifH*, and *dsrB* exhibited a positive correlation with nitrogen concentration and water content, and a negative correlation with organic carbon concentration.

**Conclusion:**

This Everglades soil metagenomic study allowed examination of wetlands biological processes and showed expected correlations between measured organic constituents and prokaryotic gene frequency. Additionally, the taxonomic profile generated gives a basis for the diversity of prokaryotic microbial life throughout the Everglades.

## Background

Wetlands serve as a major terrestrial carbon reservoir, with an estimated 20 to 30% of the global soil carbon pool, and are the largest nonanthropogenic source of atmospheric methane [1]. Microbial communities are known to play a key role in mediating carbon cycling and govern wetland greenhouse gas fluxes [2]. The Florida Everglades represent a significant wetlands area, covering 1.5 million acres, yet few studies have investigated the composition and functional potential of the resident microbial communities or the microbial processes within this ecosystem.

Previous studies within the Florida Everglades ecosystem have focused on the distribution and activity of methanogens in relation to methane cycling and emissions in the Water Conservation Area 2A (WCA-2A) [3, 4]. However, this site has experienced significant annual agricultural runoff over the past several years, resulting in a more nitrogen-limited system with an excess of phosphorous and concomitant changes in the overall microbial assemblages. Alternatively, the Loxahatchee National Wildlife Refuge has taken preventative measures in order to limit agricultural runoff, including large-scale treatment wetlands and a mandated standard of water quality [5]. Thus, the Loxahatchee Refuge represents an accessible and unperturbed system to investigate microbiome diversity and biogeochemically-relevant microbial processes.

Here we applied shotgun metagenomics to 17 wetland soil samples collected across four sites within the Loxahatchee Refuge to evaluate the taxonomic profile and functional potential of the Loxahatchee microbiome [6]. While previous studies have applied targeted gene surveys to capture methanogen populations, the present study, to our knowledge, is the first to leverage whole-genome shotgun metagenomics within the Loxahatchee Everglades ecosystem. This approach affords a unique snapshot of the resident microbial community, along with the ability to compare taxonomic and functional composition across the four sampling sites. We complemented our sequence-based analysis with bulk soil measurements of organic nitrogen and carbon, along with water content. Analysis of the 17 samples focused specifically on genes involved in metabolic pathways related to methane production (*mrcABG*), nitrogen fixation (*nifHDEK*), and sulfur reduction (*dsrAB*) due to their essential functions in major biogeochemical cycles. We hypothesize that the presence of biogeochemically-relevant marker genes (e.g., *mrcA, nifH, and dsrB)* would correlate with nutrient measurements within the samples.

## Results

**Fig. 1:**
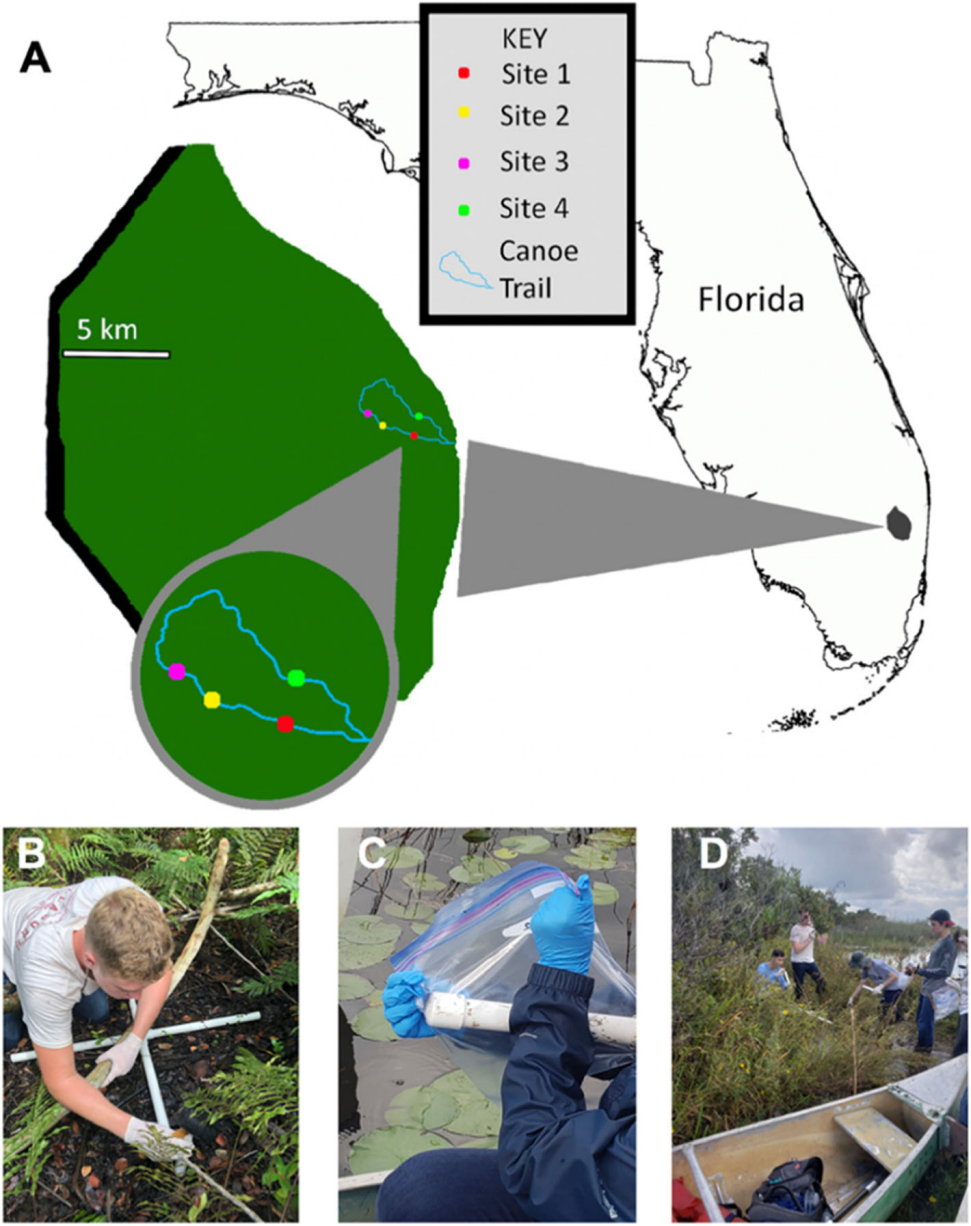
Sampling sites and collection methods. **a** Map of locations of sampling sites along trail in the Loxahatchee National Wildlife Refuge. **b** PVC pipe “X” configuration used during sample collection. **c** Method of sample packaging and storage during collection. **d** Environmental conditions present at Site 1

Shotgun metagenomic sequencing of 17 soil samples from four different sites within the Loxahatchee National Wildlife Refuge was performed using the Illumina NovaSeq platform (Fig. 1). A total of approximately 7.1 × 10^9^ filtered reads were generated, with a mean of 4.2 × 10^8^ reads per sample (Table 1). See Additional file 1: Tables S1-S3 for additional sample details.

**Table 1:** Collection coordinates and metagenomic sample data

Sites	Samples	IMG Genome ID	Gold Project ID	GOLD Analysis Project ID	NCBI BioProject ID	NCBI Biosample Accession	Genome Size Assembled (bp)	Contig Count
Site 1 (N 26.50084 W 080.23989)	Lox_Sample_1.1	3,300,032,893	Gp0356385	Ga0335069	531770	SAMN11382426	5,071,458,400	7,524,127
	Lox_Sample_1.3	3,300,032,829	Gp0356386	Ga0335070	531771	SAMN11382438	3,852,611,170	5,942,687
	Lox_Sample_1.5	3,300,032,897	Gp0356387	Ga0335071	531772	SAMN11382427	3,737,025,497	5,775,241
Site 2 (N 26.50594 W 080.25179)	Lox_Sample_2.1	3,300,032,898	Gp0356388	Ga0335072	531773	SAMN11382442	3,496,727,857	4,276,670
	Lox_Sample_2.2	3,300,033,134	Gp0356389	Ga0335073	531774	SAMN11382435	3,931,723,015	4,998,465
	Lox_Sample_2.3	3,300,032,895	Gp0356390	Ga0335074	531775	SAMN11382434	3,167,289,805	3,567,030
	Lox_Sample_2.4	3,300,032,896	Gp0356391	Ga0335075	531776	SAMN11382433	3,426,752,420	4,086,965
	Lox_Sample_2.5	3,300,032,955	Gp0356392	Ga0335076	531777	SAMN11382428	3,206,212,658	4,805,745
Site 3 (N 26.50652 W 080.25373)	Lox_Sample_3.1	3,300,033,158	Gp0356393	Ga0335077	531778	SAMN11382441	4,006,278,165	5,712,677
	Lox_Sample_3.2	3,300,032,805	Gp0356394	Ga0335078	531779	SAMN11382429	5,047,424,943	6,790,032
	Lox_Sample_3.3	3,300,032,783	Gp0356395	Ga0335079	531780	SAMN11382440	4,170,196,248	6,141,096
	Lox_Sample_3.4	3,300,032,828	Gp0356396	Ga0335080	531764	SAMN11382430	4,258,166,001	6,537,649
	Lox_Sample_3.5	3,300,032,892	Gp0356397	Ga0335081	531765	SAMN11382431	5,072,719,757	6,742,355
Site 4 (N 26.50527 W 080.23456)	Lox_Sample_4.1	3,300,032,782	Gp0356398	Ga0335082	531766	SAMN11382439	3,125,304,929	4,584,052
	Lox_Sample_4.2	3,300,032,954	Gp0356399	Ga0335083	531767	SAMN11382437	2,918,074,053	4,044,727
	Lox_Sample_4.4	3,300,033,004	Gp0356400	Ga0335084	531768	SAMN11382436	4,288,519,697	6,864,306
	Lox_Sample_4.5	3,300,032,770	Gp0356401	Ga0335085	531769	SAMN11382432	4,932,582,316	7,036,709

**Fig. 2:**
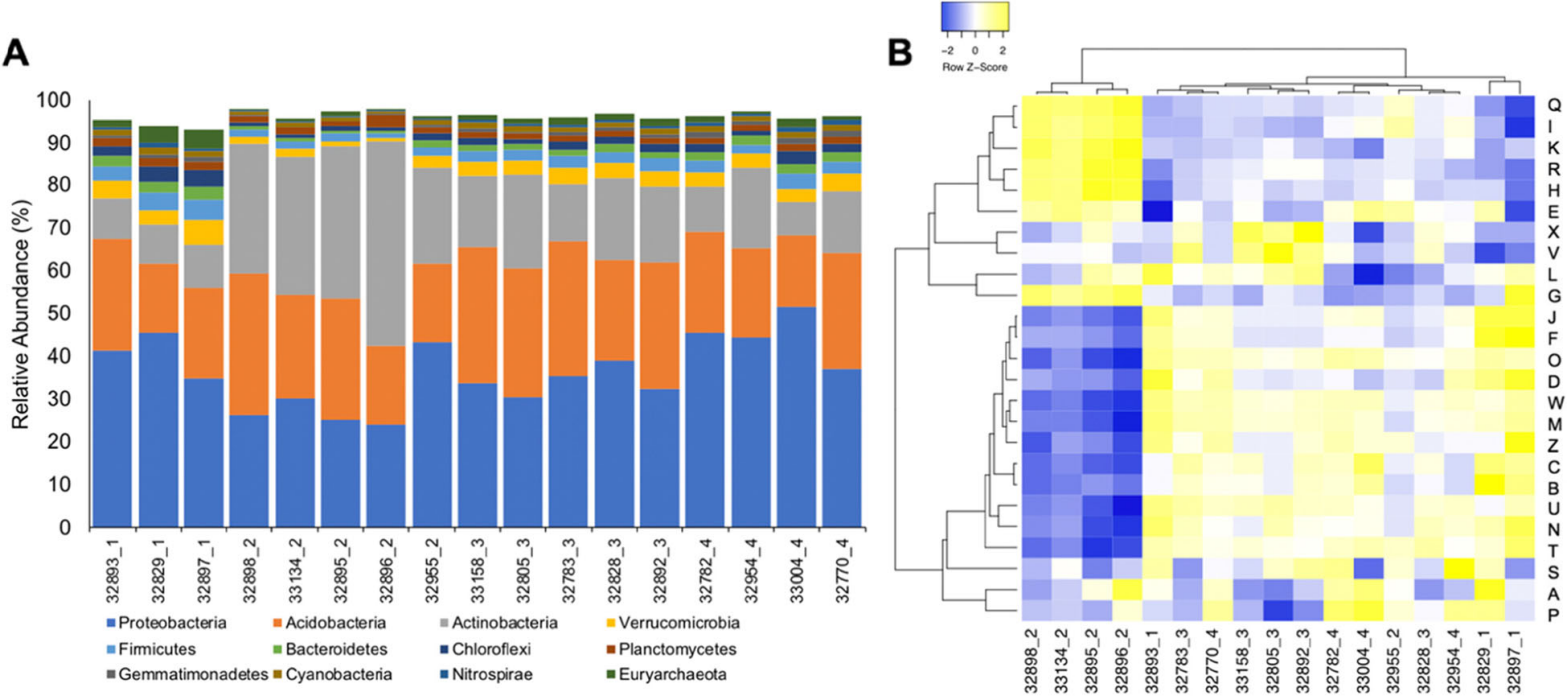
Phylogenetic and functional profile for the 17 Loxahatchee soil metagenomes. Samples are denoted by the last five digits of the IMG Genome ID, with the underscore designating the sampling sites 1–4. **a** Stacked bar charts represent relative phylum-level abundances for the most abundant phyla based on the taxonomic affiliation of the annotated proteins within each metagenome. **b** Cluster analysis of COG categories based on the relative abundances of the protein dataset within each metagenome. Heatmap is scaled by relative abundances for each row ranging from low relative abundance (blue) to high relative abundance (yellow). COG categories are as follows: A, RNA processing and modification; B, chromatin structure and dynamics; C, energy production and conversion; D, cell division, chromosome partitioning; E, amino acid transport and metabolism; F, nucleotide transport and metabolism; G, carbohydrate transport and metabolism; H, coenzyme transport and metabolism; I, lipid transport and metabolism; J, translation and biogenesis; K, transcription; L, replication, recombination, and repair; M, cell wall/membrane/envelope; N, cell motility; O, protein turnover, chaperones; P, inorganic ion transport and metabolism; Q, secondary metabolism; R, general function prediction only; S, function unknown; T, signal transduction mechanisms; U, intracellular trafficking and secretion; V, defense mechanisms; W, extracellular structures; X, Mobilome: prophages, transposons; and Z, cytoskeleton

The three most dominant bacterial phyla among all three sites were Proteobacteria, Acidobacteria, and Actinobacteria, which have been reported as common soil microorganisms (Fig. 2a) [7]. In Sites 1, 3, and 4, Proteobacteria was the most common phylum of bacteria with a relative abundance ranging from 30.4 to 51.69%. The abundance of Actinobacteria within these same sites ranged from 7.86 to 21.95%. Site 2 showed greater differences in bacterial composition, with a relatively higher abundance of Actinobacteria ranging from 22.56 to 47.75% and a lower abundance of Proteobacteria ranging from 24.16 to 43.3%. Euryarchaeota was the most common Archaea at all sites, with relative abundances ranging from 0.34 to 4.53%. A comparison of the functional profiles using the Clusters of Orthologous Groups (COGs) displayed a similar pattern, where Site 2 samples grouped together and at the exclusion of the other samples (Fig. 2b).

**Fig. 3:**
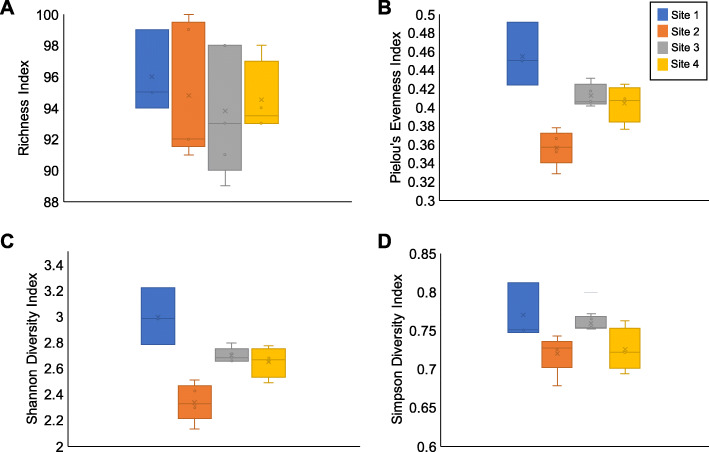
Community richness (**a**), evenness (**b**), and diversity as measured by Shannon’s and Simpson’s diversity indices (**c** and **d**) across four sites within the Loxahatchee National Wildlife Refuge

The results of alpha diversity analyses, which utilize diversity metrics, indicate a significant observable difference between Site 2 and the other sites when comparing Shannon’s diversity test, Simpson’s diversity test, and Pielou’s evenness test (Fig. 3). This is further supported by the results of the T-tests used to compare the mean average of each site to each other (see Additional file 1: Table S4). T-Tests were conducted using soil metadata which varied between samples (see Additional file 1: Table S5).

Bulk density soil samples were separately weighed, dried, and reweighed to calculate percent mass of water (see Additional file 1: Table S5). As expected in the Everglades, a high water content was found at all four sites. The soil from the least developed site, Site 1, had the highest water content (94.85%), and the soil from the most well-developed site, Site 2, had the lowest water content (74.67%). Bulk soil measurements of organic nitrogen and carbon averaged 2.6% (± 0. 5 S.D.) and 51.6% (± 4.2 S.D.), respectively (see Additional file 1: Table S5).

Normalized gene counts of biomarkers for methanogenesis (*mcrABG*), nitrogen fixation (*nifDHEK*) and dissimilatory sulfate reduction (*dsrAB*) were examined across the 17 samples. The *mcrA* gene, which encodes methyl-coenzyme M reductase (MCR) 1, is consistent through different taxa of methanogens because of its importance in methane production [3]. As the product of the *nifH* gene, nitrogenase iron protein (NIP), assists in managing the process of nitrogen fixation. As *nifH* is one of the most sequenced genes in the *nif* family across an abundance of taxa [8], the usage of *nifH* as a phylogenetic gene marker for nitrogen fixation is justified [9]. The *dsrB* gene encodes the beta subunit of sulfite reductase, which is directly involved in sulfite reduction in sulfate-reducing bacteria [10]. While gene evidence for these processes were detected in almost every sample, their relative abundances varied (see Additional file 2: Table S6). Samples from Site 1 showed the greatest abundance of these markers followed by Sites 3 and 4. Specific taxonomic lineages responsible for these processes were assessed based on the lineage assignment of the scaffolds they reside on. For methanogenesis, the most prevalent genus across samples was *Methanoregula.* This and several other known methanogen genera (e.g., *Methanocella, Methanobacterium, Methanothrix*) were detected in different samples (see Additional file 2: Table S7). Dissimilatory sulfate reduction could be attributed to members of class Beta-, Delta-, and Alphaproteobacteria and Clostridia (see Additional file 2: Table S8). Nitrogen fixation was attributed primarily to various taxa under Class Deltaproteobacteria, Nitrospira, as well as some methanogenic lineages (*Methanoregula* spp. and *Methanothrix* spp.) (see Additional file 2: Table S9). This latter observation is consistent with a previous report of potential coupling of nitrogen fixation with methanogenesis in these members in the Florida Everglades [4].

The results of a preliminary analysis using Pearson’s correlation test showed a significant correlation of *mcrA* abundance with nitrogen, carbon, and water content (percent mass of water) of the soil (*R* = 0.6401, − 0.5103, and 0.7652, respectively). Additionally, there was a significant correlation of *nifH* abundance with nitrogen, carbon, and water content (*R* = 0.7418, − 0.5057, and 0.8204, respectively). There was a significant correlation between *dsrB* abundance and nitrogen, carbon, and water contents (*R* = 0.7547, − 0.646, 0.8967, respectively). Furthermore, the percentage of genes predicted to belong to different phyla was also compared to nitrogen and carbon percentages. This analysis showed a significant correlation of Proteobacteria with nitrogen percentage in the soil (*R* = 0.6417) and no significant correlation to carbon percentage (*R* = − 0.4772). A significant correlation of Actinobacteria with nitrogen and carbon percentage was also found (*R* = − 0.8439 and *R* = 0.5432, respectively). In contrast, Acidobacteria had no significant correlation with either nitrogen or carbon content within the soil (*R* = − 0.2082 and 0.2855, respectively).

## Discussion

We found support for our hypothesis that the presence of biogeochemically-relevant marker genes (e.g., *mrcA, nifH, and dsrB)* would correlate with nutrient measurements within the samples. A Pearson’s correlation test between *mcrA* and water content yielded a positive correlation (*R* = 0.7966), which is consistent with the trend of increased methanogenesis for environments with higher water content [11]. A positive correlation between *mcrA* and nitrogen concentration (*R* = 0.6496) and a negative correlation between *mcrA* and carbon concentration (*R* = − 0.5363) was also found. The positive correlation to nitrogen concentration may be attributed to the gene’s role in nitrogen and methane cycling in wetland microbial communities [12].

Similarly, statistical tests on *nifH* abundance resulted in a significant negative correlation with total carbon content (*R* = − 0.5057) and positive correlations with both nitrogen (*R* = 0.7418) and water content (*R* = 0.8204). These results correspond to previous research that has suggested that *nifH* gene abundance is primarily impacted by factors including nitrogen concentration and microbial biomass carbon, while the negative correlation with total carbon is supported by findings that low organic matter and high microbial biomass are ideal for the presence of *nifH* [13].

The Pearson’s correlation test for *dsrB* yielded a positive correlation with nitrogen concentration (*R* = 0.7547) and water content (*R* = 0.8967) and a negative correlation with carbon concentration (*R* = − 0.646). A previous meta-analytical study using *dsrB* as a gene marker to observe a theorized sulfur cycle in wetland environments suggested a direct relationship between sulfite dissimilation and the carbon cycle due to sulfite reduction being coupled with carbon fixation in sulfate-reducing microorganisms [14]. This result corroborates the negative correlation found between the *dsrB* gene and carbon concentration within this study.

Test results showed that Proteobacteria was the most common phylum in Site 1, representing an average of 40.55% of the total assembled and annotated genes. Many Proteobacteria have symbiotic relationships with plant roots and this high concentration of Proteobacteria may be attributed to the large concentration of roots present at this site [15]. Also, as the most common phylum among Sites 3 and 4, Proteobacteria could possibly be associated with higher frequencies of the *mcrA*, *nifH*, and *dsrB* genes due to similar trends in nitrogen and carbon content. Site 2 showed greater variance of the most abundant phyla (Fig. 2a). The high presence of Actinobacteria, which has a high nitrogen-fixing capacity, correlates with data showing that *nifH* is the most common gene in Site 2 out of the four genes [16]. Similarly, Proteobacteria are also associated with the process of nitrogen fixation [17]. Acidobacteria did not significantly correlate with total carbon or nitrogen; however, due to a significant presence in the microbiome, further research regarding the phylum’s impact should be conducted.

Beta diversity test results indicated that there is a distinctive difference in taxonomy and functional capacity of Site 2 as compared to all other sites (Fig. 2). As shown in the functional profile heatmap, Site 2 samples cluster away from all the other samples, indicating dissimilarity between Site 2 and the rest of the samples. This may be due to a lower water content at Site 2, but future research should be conducted to fully determine what factors are responsible for this difference.

An important limitation in this study is the lack of replicability regarding the location of the collection sites. Since the Everglades is a fluid, shifting environment, it may be difficult to relocate the exact locations sampled in this study. Although the GPS coordinates from each of the sample sites were recorded (Table 1), the tree island areas where the samples were collected will likely drift due to different environmental factors such as rain and surrounding water level. It is possible that the same areas would not be located where the GPS coordinates indicate they were originally. However, referencing satellite imagery could help future researchers track the locations of the specific sites. Another limitation was that three out of the twenty original soil samples (Lox_Sample_1.2, Lox_Sample_1.4, Lox_Sample_4.3) did not pass the quality control stage of DNA sequencing due to low DNA content, which reduced the amount of data available for this study. It is also important to note that metagenomic sequencing finds the total number of genes that are present in the soil microorganisms but does not show how many of these genes are being expressed. Additionally, the collected soil samples did not contain enough dry matter to be tested for pH, meaning that an important aspect of metadata was lost that would have allowed for a deeper microbiome analysis. Finally, the results cannot be generalized to represent the entire Everglades region. Since all the chosen sites were located within the Loxahatchee National Wildlife Refuge, it can only be suggested that the results found within this study are representative of other sections of the Everglades.

## Conclusions

The shotgun metagenomics data described here represents, to the best of our knowledge, the only reference microbiome datasets currently available for the Loxahatchee National Wildlife Refuge within the Florida Everglades, providing valuable insight into the biogeochemical potential of the microbial communities within these wetlands ecosystems. Based on our analysis, the taxa of the sites within the Refuge were often diverse, with sites having varied taxonomic profiles. We additionally found that there is a correlation between the abundance of specific genes with both water content and the presence of different macronutrients in the soil.

Due to the extensiveness and novelty of this metagenomic study, the data generated will be extremely valuable for future researchers conducting studies within the Everglades. Particularly, researchers in the fields of conservation and methane production can use these findings as a source of information regarding methane production within the environment. Additionally, researchers studying the impacts of nitrogen pollution on the Everglades can use these findings to predict how the microbiome changes between locations and observe how the data generated compares to other findings. Researchers could also attempt to replicate this metagenomic analysis in other locations using similar research methods, which would allow comparisons to be conducted between the soil metagenomes. Future studies on the Everglades soil microbiome could eventually lead to crucial discoveries in the fields of biofuel production and methane regulation.

## Methods

### Sample collection and processing

Soil samples were collected at the Arthur R. Marshall Loxahatchee National Wildlife Refuge in Palm Beach County, Florida on November 11th, 2018. The Loxahatchee National Wildlife Refuge, established in 1951, is an approximately 143,954 acre wildlife reserve in Palm Beach County, Florida. It is the only remaining section of the Everglades in Palm Beach County and is surrounded by farmland to the west, urban housing to the east, and the Everglades National Park to the south. This area also serves as a drainage point of Lake Okeechobee to the north. The Loxahatchee ecosystem provides a habitat for over 250 species of birds, as well as dozens of mammals, reptiles, and amphibians, including multiple endangered species [1]. Four sample sites were chosen based on specific criteria, including location accessibility and the ability to extract samples.

The sites shared many similar aspects; however, not every area had identical conditions. Sites 2 and 3 were very distinct tree islands (dirt, roots, and organic matter from trees and plants that form a mass of soil and vegetation above the water level). Site 2 had a prominent red bay tree (*Persea borbonia*) population, and Site 3 had an overgrowth of Old World Climbing Fern (*Lygodium microphyllum*). On the other hand, Sites 1 and 4 were not as well established, with Site 1 being a floating grass marsh with an abundance of Leavenworth’s tickseed (*Coreopsis leavenworth*) and Site 4 being covered in dense, muddy sawgrass (*Cladium jamaicense*). Sites 2 and 4 consisted of very dry and silt-like soil, whereas Sites 1 and 3 were covered with wet and mud-like soil with a higher concentration of roots and vegetation. Sites 1 and 3 appeared to be established more recently and were very close to, or below, the water table of the area. This contrasted with Sites 2 and 4, which were at least half a meter over the water table.

For the collection process, evenly sized, sterilized, and capped PVC coring devices were constructed in order to extract soil samples. Five samples were collected from all four sites, for a total of 20 samples. The samples were collected in an “X” pattern where a sample was taken at each end of the “X” configuration as well as the point in the center where the PVC pipes met. The distance of the four end samples from the center sample was approximately half a meter (Fig. 1b). Each core was used to take samples from the top 15 to 20 cm of soil. Additional soil was taken from the third soil core location at each site to calculate bulk density. For this additional soil collection, a container with a volume of approximately 285 ml was filled with topsoil. Proper precautions were taken to keep samples uncontaminated during transport (Fig. 1c). Prior to DNA extraction, each sample was sifted through sanitized mesh in order to remove material such as roots and plant matter, leaving only the targeted soil.

### DNA extraction and sequencing

DNA from each soil sample was extracted within 12 h of sample collection using the QIAGEN DNeasy® PowerSoil® Kit (QIAGEN, Hilden, Germany). After extraction, the DNA samples were stored and frozen at − 20 °C until being sent on dry ice to the Joint Genome Institute (JGI) in Walnut Creek, California approximately 24 h later. The JGI was responsible for performing all DNA sequencing. Standard protocols for shotgun metagenomic sequencing were followed and performed on the Illumina NovaSeq 6000 platform (Illumina, San Diego, CA, USA). NovaSeq sequencing generated 7.1 × 10^9^ filtered reads with 6.8 × 10^10^ total bp.

### Metagenome quality control, assembly, and annotation

The JGI utilized the BBTools software package to filter the reads as well as BFC (version r181) to correct the sequencing errors in the Illumina short reads. BBDuk was utilized to remove contaminants from the samples, to trim reads with extraneous adapters, and to remove reads with a length of less than 51 bp. Metagenome assembly was performed using metaSPAdes (version 3.13.0). The filtered read set was mapped to the final assembly and coverage information was generated using bbmap (Version 38.25) using default parameters, with the exception of ambiguous = random. The processing pipeline used was jgi_meta_run.py (version 2.0.1). In Quality Control, three samples (Lox_Sample_1.2, Lox_Sample_1.4, and Lox_Sample_4.3) were discontinued because they failed to meet the minimum amount of DNA concentration required to move to sequencing. Assembled metagenomes were processed through the DOE-JGI Metagenome Annotation Pipeline and loaded into the Integrated Microbial Genome & Microbiomes platform (IMG/M) [18]. Sample metadata is available through the Genomes OnLine Database (GOLD) [19].

### Soil analysis

Sifted soil samples (~ 10 g) were sent to Stable Isotopes for Biosphere Science Laboratory (Texas A&M University, Department of Ecosystem Science and Management. https://sibs.tamu.edu/) for analysis of organic nitrogen and carbon concentrations. The soil samples were dried at 60 °C in an oven for 3 days to the constant weights, and ground to fine powder using Retesch Oscillating Mixer Mill MM400 (Haan, Germany). The samples were analyzed using the Costech Elemental Combustion System (Costech Analytical Technologies, Santa Clarita, CA, USA) coupled to a Thermo Conflo IV Interface (Thermo Fisher Scientific, Waltham, MA, USA) and a Thermo Scientific Delta V Advantage Stable Isotope Mass Spectrometer (Thermo Fisher Scientific, Waltham, MA, USA). The NIST plant standard Apple1515 was used to calculate the Nitrogen and Carbon concentrations (%).

Sample bulk density from each location was determined at Boca Raton Community High School the (Boca Raton, Florida). Samples from each site were separately weighed after collection, dried at 80 °C for 7 days to constant weights, and reweighed to determine their percent mass of water.

### Statistical analyses used

Statistical analysis of the 17 samples focused specifically on genes involved in metabolic pathways related to methane production (mrcABG), nitrogen fixation (nifHDEK), and sulfur reduction (dsrAB) due to their essential functions in major biogeochemical cycles. The genes in question were identified using their KEGG Orthology (KO) terms to account for the diversity of the enzymes, and included *mcrA* (K00399), *nifH* (K02588), and *dsrB* (K11181). Using the JGI’s Integrated Microbial Genomes & Microbiomes (IMG/M) platform (https://img.jgi.doe.gov, version 5.0), the abundance of each gene was found by matching predicted genes with reference genes [19]. Data were normalized by using the number of estimated gene copies for each gene involved in the study divided by the total number of genes per metagenome. This took into consideration differences in sequencing coverage between samples, making the gene counts directly comparable. Finally, the relative abundance for each gene was compared to the water content, nitrogen content, and carbon content using Pearson’s linear correlation to determine statistical significance. Spearman’s rank correlation test was performed in order to validate the results from Pearson’s linear correlation test.

The phylogenetic and functional distribution of genes in the samples was determined using the JGI’s Integrated Microbial Genomes & Microbiomes (IMG/M) platform (https://img.jgi.doe.gov, version 5.0) [19]. Phylogenetic distribution was based off of best BLAST hits of potential protein coding genes. A threshold of > 30% BLAST percent identity against the non-redundant reference genome database was used to assign taxonomy. The phyla with the three highest percentages of genes were then compared to nitrogen and carbon content in the soil using Pearson’s linear correlation to determine significance.

Alpha and beta diversity analyses were obtained using Scikit Bio (version 0.54) in python 3.6.8. For taxonomic profiling, the estimated gene copies for each sample were used to normalize the data, which were attained through IMG/M using a blast identity of at least 30%.

## Supplementary information

**Additional file 1: Tables S1-S5.** Containing metadata from sequencing, assembly information, potential contaminants, diversity tests, and soil content.

**Additional file 2: Tables S6-S9.** Containing biomarker gene counts, taxonomic data for methanogens, sulfate reducers, and nitrogen fixers.

## Data Availability

For shotgun metagenomics library and data, refer to the JGI Genome Portal Page: https://genome.jgi.doe.gov/portal/SoimetHighSchool/SoimetHighSchool.info.html. Table 1 displays JGI IMG/M [19,20] Genome ID number, GOLD Analysis Project ID, and NCBI Accessions, and NCBI Accessions of each sample. Full metagenomic data and sequences can be found by searching IMG/M/M (https://img.jgi.doe.gov/cgi-bin/m/main.cgi) for the Genome IDs found on Table 1.

## Abbreviations

BLAST: Basic Local Alignment Search Tool
bp: Base pair
CA: California
DNA: Deoxyribonucleic acid
GOLD: Genomes OnLine Database
GPS: Global Positioning System
IMG/M: Integrated Microbial Genomes & Microbiomes
JGI: Joint Genome Institute
KO: KEGG Orthology
Lox: Loxahatchee
MA: Massachusetts
MDS: Multidimensional scaling
NCBI: National Center for Biotechnology Information
NIST: National Institute of Standards and Technology
PCA: Principal component analysis
PVC: Polyvinyl chloride
USA: United States of America
