# Supplementary material for: Shotgun metagenomic analysis of microbial communities from the Loxahatchee nature preserve in the Florida Everglades
Source: Environ Microbiome. 2020 Jan 21;15:2. doi: 10.1186/s40793-019-0352-4 (PMC8067648; doi:10.1186/s40793-019-0352-4)
Supplement: Supplementary file 1 — Additional file 1: Tables S1-S5. Containing metadata from sequencing, assembly information, potential contaminants, diversity tests, and soil content. [file 40793_2019_352_MOESM1_ESM.docx]

Supplementary Materials for

**Shotgun metagenomic analysis of microbial communities from the Loxahatchee Nature Preserve in the Florida Everglades**

B. Abraham, D. Caglayan, N. Carrillo, M. Chapman, C. Hagan, S. Hansen, R. Jeanty, A. Klimczak, M. Klingler, T. Kutcher, S. Levy, A. Millard-Bruzos, T. Moore, D Prentice, M. Prescott, R. Roehm, J. Rose, M. Yin, A. Hyodo, K. Lail, C. Daum, A. Clum, A. Copeland, R. Seshadri, T. Glavina del Rio, E. Eloe-Fadrosh, J. Benskin

Correspondence to: jonathan.benskin@palmbeachschools.org

Table S1: Sample metadata from shotgun sequencing.

| **Sample Name** | **Platform** | **Instrument** | **Read Length** | **Read Count** | **Filtered Read Count** | **Percent of Reads ≥ Q30 Average** | **Average Base Quality Score** | **Insert Size** | **Insert Size Mode** | **Percent Read Pairs Merged** | **Q20 Read Length, Read 1** | **Q20 Read Length, Read 2** | **Read GC** |
| --- | --- | --- | --- | --- | --- | --- | --- | --- | --- | --- | --- | --- | --- |
| Lox Sample 1.1 | Illumina | NovaSeq | 2x151 bp | 522,070,234 | 514,360,440 | 95.59% | 36.24 ± 3.81 | 241.5 ± 40.0 | 287 | 21.47% | 150 bp | 150 bp | 61.15 ± 9.19% |
| Lox Sample 1.3 | Illumina | NovaSeq | 2x151 bp | 447,512,718 | 433,445,750 | 95.35% | 36.19 ± 3.96 | 241.6 ± 40.8 | 287 | 14.51% | 150 bp | 150 bp | 60.76 ± 9.55% |
| Lox Sample 1.5 | Illumina | NovaSeq | 2x151 bp | 410,632,630 | 394,531,032 | 95.25% | 36.17 ± 4.00 | 245.5 ± 37.5 | 287 | 17.34% | 150 bp | 150 bp | 60.73 ± 9.53% |
| Lox Sample 2.1 | Illumina | NovaSeq | 2x151 bp | 413,738,464 | 405,667,496 | 95.64% | 36.24 ± 3.85 | 248.5 ± 35.9 | 286 | 16.14% | 150 bp | 150 bp | 62.29 ± 9.00% |
| Lox Sample 2.2 | Illumina | NovaSeq | 2x151 bp | 431,274,764 | 421,030,614 | 95.30% | 36.17 ± 4.01 | 247.4 ± 36.1 | 286 | 15.39% | 150 bp | 150 bp | 247.4 ± 36.1 |
| Lox Sample 2.3 | Illumina | NovaSeq | 2x151 bp | 390,401,006 | 382,325,580 | 95.45% | 36.20 ± 3.93 | 248.5 ± 35.1 | 286 | 17.42% | 150 bp | 150 bp | 62.92 ± 9.08% |
| Lox Sample 2.4 | Illumina | NovaSeq | 2x151 bp | 424,395,002 | 415,646,988 | 95.58% | 36.22 ± 3.90 | 248.6 ± 35.0 | 287 | 16.70% | 150 bp | 150 bp | 63.73 ± 9.25% |
| Lox Sample 2.5 | Illumina | NovaSeq | 2x151 bp | 355,547,126 | 355,547,126 | 95.38% | 36.18 ± 3.99 | 246.6 ± 36.3 | 283 | 19.05% | 150 bp | 150 bp | 62.71 ± 8.78% |
| Lox Sample 3.1 | Illumina | NovaSeq | 2x151 bp | 383,074,976 | 380,904,536 | 95.74% | 36.27 ± 3.73 | 248.6 ± 34.4 | 285 | 21.84% | 150 bp | 150 bp | 62.49 ± 7.61% |
| Lox Sample 3.2 | Illumina | NovaSeq | 2x151 bp | 506,491,070 | 501,896,854 | 95.84% | 36.28 ± 3.72 | 247.7 ± 35.3 | 286 | 22.19% | 150 bp | 150 bp | 62.20 ± 8.00% |
| Lox Sample 3.3 | Illumina | NovaSeq | 2x151 bp | 398,051,504 | 393,458,660 | 95.64% | 36.24 ± 3.83 | 248.4 ± 34.8 | 286 | 20.15% | 150 bp | 150 bp | 61.72 ± 8.04% |
| Lox Sample 3.4 | Illumina | NovaSeq | 2x151 bp | 412,383,228 | 407,631,926 | 95.69% | 36.26 ± 3.79 | 248.6 ± 34.7 | 286 | 19.71% | 150 bp | 150 bp | 62.49 ± 8.07% |
| Lox Sample 3.5 | Illumina | NovaSeq | 2x151 bp | 490,564,808 | 485,969,802 | 95.82% | 36.28 ± 3.71 | 248.7 ± 34.3 | 284 | 20.11% | 150 bp | 150 bp | 62.08 ± 7.78% |
| Lox Sample 4.1 | Illumina | NovaSeq | 2x151 bp | 337,679,286 | 333,805,618 | 94.95% | 36.13 ± 4.06 | 245.6 ± 36.2 | 287 | 22.09% | 150 bp | 150 bp | 62.40 ± 8.55% |
| Lox Sample 4.2 | Illumina | NovaSeq | 2x151 bp | 318,916,728 | 314,631,176 | 95.62% | 36.24 ± 3.84 | 246.9 ± 35.5 | 284 | 19.55% | 150 bp | 150 bp | 62.57 ± 8.28% |
| Lox Sample 4.4 | Illumina | NovaSeq | 2x151 bp | 412,287,960 | 408,850,934 | 95.85% | 36.28 ± 3.71 | 244.7 ± 36.7 | 281 | 23.13% | 150 bp | 150 bp | 62.91 ± 8.51% |
| Lox Sample 4.5 | Illumina | NovaSeq | 2x151 bp | 517,796,346 | 514,390,536 | 96.10% | 36.34 ± 3.55 | 249.3 ± 33.3 | 285 | 24.77% | 150 bp | 150 bp | 62.40 ± 8.04% |

**Table S2: Reads Matching Potential Contamination.**

| **Sample Name** | **Adapters** | **Adapters (first 50bp only)** | **DNA Spike-ins** | **RNA Spike-ins** | **Fosmid Vector** | **PhiX** | **Non Synthetic** | **Mitochondria** | **Chloroplast** | **rRNA** |
| --- | --- | --- | --- | --- | --- | --- | --- | --- | --- | --- |
| Lox Sample 1.1 | 0.90% | 0.62% | 0.00% | 0.00% | 0.00% | 0.00% | 0.00% | 3.88% | 4.06% | 0.63% |
| Lox Sample 1.3 | 1.26% | 1.05% | 0.00% | 0.00% | 0.00% | 0.00% | 0.00% | 5.65% | 5.77% | 0.71% |
| Lox Sample 1.5 | 1.15% | 0.96% | 0.00% | 0.00% | 0.00% | 0.00% | 0.00% | 6.51% | 6.62% | 0.67% |
| Lox Sample 2.1 | 1.42% | 1.31% | 0.00% | 0.00% | 0.00% | 0.00% | 0.00% | 4.28% | 4.21% | 0.80% |
| Lox Sample 2.2 | 1.58% | 1.46% | 0.00% | 0.00% | 0.00% | 0.00% | 0.00% | 4.58% | 4.49% | 0.79% |
| Lox Sample 2.3 | 1.32% | 1.22% | 0.00% | 0.00% | 0.00% | 0.00% | 0.00% | 4.21% | 4.09% | 0.81% |
| Lox Sample 2.4 | 1.48% | 1.38% | 0.00% | 0.00% | 0.00% | 0.00% | 0.00% | 4.44% | 4.31% | 0.84% |
| Lox Sample 2.5 | 1.88% | 1.75% | 0.00% | 0.00% | 0.00% | 0.00% | 0.00% | 4.25% | 4.23% | 0.88% |
| Lox Sample 3.1 | 0.50% | 0.40% | 0.00% | 0.00% | 0.00% | 0.00% | 0.00% | 2.04% | 1.99% | 0.51% |
| Lox Sample 3.2 | 0.85% | 0.73% | 0.00% | 0.00% | 0.00% | 4e–05% | 0.00% | 2.68% | 2.60% | 0.56% |
| Lox Sample 3.3 | 0.98% | 0.88% | 0.00% | 0.00% | 5e–05% | 0.00% | 0.00% | 2.99% | 2.95% | 0.66% |
| Lox Sample 3.4 | 0.96% | 0.85% | 0.00% | 0.00% | 0.00% | 0.00% | 0.00% | 2.80% | 2.76% | 0.60% |
| Lox Sample 3.5 | 0.80% | 0.71% | 0.00% | 0.00% | 0.00% | 4e–05% | 4e–05% | 2.48% | 2.41% | 0.52% |
| Lox Sample 4.1 | 1.15% | 1.01% | 0.00% | 0.00% | 0.00% | 0.00% | 0.00% | 3.06% | 3.05% | 0.71% |
| Lox Sample 4.2 | 1.10% | 0.99% | 0.00% | 0.00% | 0.00% | 0.00% | 6e–05% | 3.20% | 3.17% | 0.72% |
| Lox Sample 4.4 | 0.72% | 0.59% | 0.00% | 0.00% | 0.00% | 5e–05% | 0.00% | 2.58% | 2.59% | 0.54% |
| Lox Sample 4.5 | 0.44% | 0.34% | 0.00% | 0.00% | 0.00% | 0.00% | 0.00% | 2.32% | 2.27% | 0.54% |

**Table S3: Assembly Information.**

| **Sample Name** | **Contig Count** | **Total Length** | **N50** | **Max Contig Length** | **L50** | **L90** | **Ratio of reads mapped to contigs** | **Ratio of reads mapped to contigs ≥ 10kb** |
| --- | --- | --- | --- | --- | --- | --- | --- | --- |
| Lox Sample 1.1 | 7,524,127 | 5,073,890,161 bp | 1,385,399 | 966,835 | 762 | 1,001 | 60.40% | 11.00% |
| Lox Sample 1.3 | 5,942,687 | 3,854,319,575 bp | 1,162,931 | 778,343 | 709 | 633 | 47.40% | 6.20% |
| Lox Sample 1.5 | 5,775,241 | 3,738,543,218 bp | 1,175,086 | 881,419 | 713 | 610 | 50.20% | 6.20% |
| Lox Sample 2.1 | 4,276,670 | 3,498,326,906 bp | 590,918 | 1,212,572 | 1,120 | 780 | 72.10% | 16.70% |
| Lox Sample 2.2 | 4,998,465 | 3,933,254,154 bp | 779,684 | 1,136,497 | 1,030 | 925 | 69.10% | 11.90% |
| Lox Sample 2.3 | 3,567,030 | 3,168,631,094 bp | 465,707 | 829,527 | 1,332 | 731 | 80.60% | 17.30% |
| Lox Sample 2.4 | 4,086,965 | 3,428,446,172 bp | 537,217 | 1,118,386 | 1,181 | 780 | 75.40% | 16.20% |
| Lox Sample 2.5 | 4,805,745 | 3,208,035,624 bp | 919,469 | 478,418 | 757 | 506 | 50.40% | 5.50% |
| Lox Sample 3.1 | 5,712,677 | 4,007,317,427 bp | 1,024,190 | 1,203,259 | 822 | 761 | 62.00% | 8.20% |
| Lox Sample 3.2 | 6,790,032 | 5,048,854,196 bp | 1,099,745 | 1,101,837 | 930 | 1,255 | 71.10% | 12.50% |
| Lox Sample 3.3 | 6,141,096 | 4,171,278,819 bp | 1,171,313 | 812,264 | 775 | 767 | 56.70% | 6.60% |
| Lox Sample 3.4 | 6,537,649 | 4,259,582,075 bp | 1,303,430 | 618,498 | 726 | 739 | 53.20% | 5.00% |
| Lox Sample 3.5 | 6,742,355 | 5,073,855,621 bp | 1,069,897 | 1,322,185 | 933 | 1,285 | 70.70% | 13.70% |
| Lox Sample 4.1 | 4,584,052 | 3,126,416,126 bp | 834,903 | 436,316 | 784 | 494 | 54.20% | 7.50% |
| Lox Sample 4.2 | 4,044,727 | 2,918,987,207 bp | 656,909 | 1,646,909 | 857 | 464 | 55.20% | 11.40% |
| Lox Sample 4.4 | 6,864,306 | 4,290,243,432 bp | 1,462,192 | 144,519 | 677 | 689 | 50.80% | 4.30% |
| Lox Sample 4.5 | 7,036,709 | 4,934,283,054 bp | 1,169,774 | 3,096,523 | 820 | 1,057 | 68.50% | 20.50% |

Table S4: t-Tests for different diversity tests.

|  | **Richness** | | |  | **Shannon's** | | |  | **Simpson's** | | |  | **Peilou's** | | |
| --- | --- | --- | --- | --- | --- | --- | --- | --- | --- | --- | --- | --- | --- | --- | --- |
| **Site 1 v. Site 2** | F-Test Two-Sample for Variances | | |  | F-Test Two-Sample for Variances | | |  | F-Test Two-Sample for Variances | | |  | F-Test Two-Sample for Variances | | |
|  |  |  |  |  |  |  |  |  |  |  |  |  |  |  |  |
|  |  | Variable 1 | Variable 2 |  |  | Variable 1 | Variable 2 |  |  | Variable 1 | Variable 2 |  |  | Variable 1 | Variable 2 |
|  | Mean | 96 | 94.8 |  | Mean | 2.99518714 | 2.33809798 |  | Mean | 0.76986687 | 0.7203672 |  | Mean | 0.4549209 | 0.35598724 |
|  | Variance | 7 | 18.7 |  | Variance | 0.0472816 | 0.02007615 |  | Variance | 0.00132693 | 0.00059764 |  | Variance | 0.00114995 | 0.00034211 |
|  | Observations | 3 | 5 |  | Observations | 3 | 5 |  | Observations | 3 | 5 |  | Observations | 3 | 5 |
|  | df | 2 | 4 |  | df | 2 | 4 |  | df | 2 | 4 |  | df | 2 | 4 |
|  | F | 0.37433155 |  |  | F | 2.3551125 |  |  | F | 2.22029512 |  |  | F | 3.36139115 |  |
|  | P(F<=f) one-tail | 0.29045938 |  |  | P(F<=f) one-tail | 0.21089255 |  |  | P(F<=f) one-tail | 0.22458169 |  |  | P(F<=f) one-tail | 0.13915698 |  |
|  | F Critical one-tail | 0.0519567 |  |  | F Critical one-tail | 6.94427191 |  |  | F Critical one-tail | 6.94427191 |  |  | F Critical one-tail | 6.94427191 |  |
|  |  |  |  |  |  |  |  |  |  |  |  |  |  |  |  |
| **Site 1 v. Site 3** | F-Test Two-Sample for Variances | | |  | F-Test Two-Sample for Variances | | |  | F-Test Two-Sample for Variances | | |  | F-Test Two-Sample for Variances | | |
|  |  |  |  |  |  |  |  |  |  |  |  |  |  |  |  |
|  |  | Variable 1 | Variable 2 |  |  | Variable 1 | Variable 2 |  |  | Variable 1 | Variable 2 |  |  | Variable 1 | Variable 2 |
|  | Mean | 96 | 93.8 |  | Mean | 2.99518714 | 2.70002298 |  | Mean | 0.76986687 | 0.75908476 |  | Mean | 0.4549209 | 0.41227235 |
|  | Variance | 7 | 16.7 |  | Variance | 0.0472816 | 0.00328982 |  | Variance | 0.00132693 | 7.78E-05 |  | Variance | 0.00114995 | 0.00014632 |
|  | Observations | 3 | 5 |  | Observations | 3 | 5 |  | Observations | 3 | 5 |  | Observations | 3 | 5 |
|  | df | 2 | 4 |  | df | 2 | 4 |  | df | 2 | 4 |  | df | 2 | 4 |
|  | F | 0.41916168 |  |  | F | 14.3721047 |  |  | F | 17.0561836 |  |  | F | 7.85920117 |  |
|  | P(F<=f) one-tail | 0.31651309 |  |  | P(F<=f) one-tail | 0.01492282 |  |  | P(F<=f) one-tail | 0.01101509 |  |  | P(F<=f) one-tail | 0.04115063 |  |
|  | F Critical one-tail | 0.0519567 |  |  | F Critical one-tail | 6.94427191 |  |  | F Critical one-tail | 6.94427191 |  |  | F Critical one-tail | 6.94427191 |  |
|  |  |  |  |  |  |  |  |  |  |  |  |  |  |  |  |
| **Site 1 v. Site 4** | F-Test Two-Sample for Variances | | |  | F-Test Two-Sample for Variances | | |  | F-Test Two-Sample for Variances | | |  | F-Test Two-Sample for Variances | | |
|  |  |  |  |  |  |  |  |  |  |  |  |  |  |  |  |
|  |  | Variable 1 | Variable 2 |  |  | Variable 1 | Variable 2 |  |  | Variable 1 | Variable 2 |  |  | Variable 1 | Variable 2 |
|  | Mean | 96 | 94.5 |  | Mean | 2.99514328 | 2.64967864 |  | Mean | 0.76986687 | 0.72506256 |  | Mean | 0.4549209 | 0.40387845 |
|  | Variance | 7 | 5.66666667 |  | Variance | 0.04728652 | 0.01408726 |  | Variance | 0.00132693 | 0.00080239 |  | Variance | 0.00114995 | 0.00040117 |
|  | Observations | 3 | 4 |  | Observations | 3 | 4 |  | Observations | 3 | 4 |  | Observations | 3 | 4 |
|  | df | 2 | 3 |  | df | 2 | 3 |  | df | 2 | 3 |  | df | 2 | 3 |
|  | F | 1.23529412 |  |  | F | 3.3566878 |  |  | F | 1.65372615 |  |  | F | 2.86647503 |  |
|  | P(F<=f) one-tail | 0.40609799 |  |  | P(F<=f) one-tail | 0.1716432 |  |  | P(F<=f) one-tail | 0.32802045 |  |  | P(F<=f) one-tail | 0.2013448 |  |
|  | F Critical one-tail | 9.5520945 |  |  | F Critical one-tail | 9.5520945 |  |  | F Critical one-tail | 9.5520945 |  |  | F Critical one-tail | 9.5520945 |  |
|  |  |  |  |  |  |  |  |  |  |  |  |  |  |  |  |
|  |  |  |  |  |  |  |  |  |  |  |  |  |  |  |  |
| **Site 2 v. Site 3** | F-Test Two-Sample for Variances | | |  | F-Test Two-Sample for Variances | | |  | F-Test Two-Sample for Variances | | |  | F-Test Two-Sample for Variances | | |
|  |  |  |  |  |  |  |  |  |  |  |  |  |  |  |  |
|  |  | Variable 1 | Variable 2 |  |  | Variable 1 | Variable 2 |  |  | Variable 1 | Variable 2 |  |  | Variable 1 | Variable 2 |
|  | Mean | 94.8 | 93.8 |  | Mean | 2.33809798 | 2.70002298 |  | Mean | 0.7203672 | 0.75908476 |  | Mean | 0.35598724 | 0.41227235 |
|  | Variance | 18.7 | 16.7 |  | Variance | 0.02007615 | 0.00328982 |  | Variance | 0.00059764 | 7.78E-05 |  | Variance | 0.00034211 | 0.00014632 |
|  | Observations | 5 | 5 |  | Observations | 5 | 5 |  | Observations | 5 | 5 |  | Observations | 5 | 5 |
|  | df | 4 | 4 |  | df | 4 | 4 |  | df | 4 | 4 |  | df | 4 | 4 |
|  | F | 1.11976048 |  |  | F | 6.10251303 |  |  | F | 7.68194438 |  |  | F | 2.33807992 |  |
|  | P(F<=f) one-tail | 0.4576722 |  |  | P(F<=f) one-tail | 0.05388784 |  |  | P(F<=f) one-tail | 0.0367442 |  |  | P(F<=f) one-tail | 0.21546272 |  |
|  | F Critical one-tail | 6.38823291 |  |  | F Critical one-tail | 6.38823291 |  |  | F Critical one-tail | 6.38823291 |  |  | F Critical one-tail | 6.38823291 |  |
|  |  |  |  |  |  |  |  |  |  |  |  |  |  |  |  |
| **Site 2 v. Site 4** | F-Test Two-Sample for Variances | | |  | F-Test Two-Sample for Variances | | |  | F-Test Two-Sample for Variances | | |  | F-Test Two-Sample for Variances | | |
|  |  |  |  |  |  |  |  |  |  |  |  |  |  |  |  |
|  |  | Variable 1 | Variable 2 |  |  | Variable 1 | Variable 2 |  |  | Variable 1 | Variable 2 |  |  | Variable 1 | Variable 2 |
|  | Mean | 94.8 | 94.5 |  | Mean | 2.33809798 | 2.64971279 |  | Mean | 0.7203672 | 0.72506256 |  | Mean | 0.35598724 | 0.40387845 |
|  | Variance | 18.7 | 5.66666667 |  | Variance | 0.02007615 | 0.01407272 |  | Variance | 0.00059764 | 0.00080239 |  | Variance | 0.00034211 | 0.00040117 |
|  | Observations | 5 | 4 |  | Observations | 5 | 4 |  | Observations | 5 | 4 |  | Observations | 5 | 4 |
|  | df | 4 | 3 |  | df | 4 | 3 |  | df | 4 | 3 |  | df | 4 | 3 |
|  | F | 3.3 |  |  | F | 1.42660032 |  |  | F | 0.74482268 |  |  | F | 0.8527645 |  |
|  | P(F<=f) one-tail | 0.17709115 |  |  | P(F<=f) one-tail | 0.40112248 |  |  | P(F<=f) one-tail | 0.37898756 |  |  | P(F<=f) one-tail | 0.42442992 |  |
|  | F Critical one-tail | 9.11718225 |  |  | F Critical one-tail | 9.11718225 |  |  | F Critical one-tail | 0.15171325 |  |  | F Critical one-tail | 0.15171325 |  |
|  |  |  |  |  |  |  |  |  |  |  |  |  |  |  |  |
| **Site 3 v. Site 4** | F-Test Two-Sample for Variances | | |  | F-Test Two-Sample for Variances | | |  | F-Test Two-Sample for Variances | | |  | F-Test Two-Sample for Variances | | |
|  |  |  |  |  |  |  |  |  |  |  |  |  |  |  |  |
|  |  | Variable 1 | Variable 2 |  |  | Variable 1 | Variable 2 |  |  | Variable 1 | Variable 2 |  |  | Variable 1 | Variable 2 |
|  | Mean | 93.8 | 94.5 |  | Mean | 2.70002298 | 2.64971279 |  | Mean | 0.75908476 | 0.72506256 |  | Mean | 0.41227235 | 0.40387845 |
|  | Variance | 16.7 | 5.66666667 |  | Variance | 0.00328982 | 0.01407272 |  | Variance | 7.78E-05 | 0.00080239 |  | Variance | 0.00014632 | 0.00040117 |
|  | Observations | 5 | 4 |  | Observations | 5 | 4 |  | Observations | 5 | 4 |  | Observations | 5 | 4 |
|  | df | 4 | 3 |  | df | 4 | 3 |  | df | 4 | 3 |  | df | 4 | 3 |
|  | F | 2.94705882 |  |  | F | 0.2337726 |  |  | F | 0.09695757 |  |  | F | 0.36472855 |  |
|  | P(F<=f) one-tail | 0.2006232 |  |  | P(F<=f) one-tail | 0.09707563 |  |  | P(F<=f) one-tail | 0.0236133 |  |  | P(F<=f) one-tail | 0.1772774 |  |
|  | F Critical one-tail | 9.11718225 |  |  | F Critical one-tail | 0.15171325 |  |  | F Critical one-tail | 0.15171325 |  |  | F Critical one-tail | 0.15171325 |  |

**Table S5: Soil metadata for each sample.**

| **Site Name** | **%N** | **%C** | **Water %** |
| --- | --- | --- | --- |
| Lox_sample_1.1 | 3.2 | 50 | 94.85 |
| Lox_smaple_1.3 | 2.6 | 38.3 | 94.85 |
| Lox_sample_1.5 | 3.4 | 52 | 94.85 |
| Lox_sample_2.1 | 2.1 | 54.1 | 74.67 |
| Lox_sample_2.2 | 2.3 | 60.5 | 74.67 |
| Lox_sample_2.3 | 2 | 53.5 | 74.67 |
| Lox_sample_2.4 | 1.8 | 54.5 | 74.67 |
| Lox_sample_2.5 | 2.3 | 52.4 | 74.67 |
| Lox_sample_3.1 | 2.4 | 51.1 | 87.36 |
| Lox_sample_3.2 | 2.3 | 49.7 | 87.36 |
| Lox_sample_3.3 | 2.6 | 52.8 | 87.36 |
| Lox_sample_3.4 | 2.1 | 50.9 | 87.36 |
| Lox_sample_3.5 | 2.6 | 51.1 | 87.36 |
| Lox_sample_4.1 | 3 | 51.8 | 86.27 |
| Lox_sample_4.2 | 2.7 | 51.8 | 86.27 |
| Lox_sample_4.4 | 3.1 | 50.9 | 86.27 |
| Lox_sample_4.5 | 2.9 | 51.7 | 86.27 |
